# Genome-wide transcriptional analysis of T cell activation reveals differential gene expression associated with psoriasis

**DOI:** 10.1186/1471-2164-14-825

**Published:** 2013-11-23

**Authors:** Nuria Palau, Antonio Julià, Carlos Ferrándiz, Lluís Puig, Eduardo Fonseca, Emilia Fernández, María López-Lasanta, Raül Tortosa, Sara Marsal

**Affiliations:** Rheumatology Research Group, Vall d’Hebron Research Institute, Barcelona, 08035 Spain; Dermatology Service, Hospital Universitari Germans Trias i Pujol, Badalona, 08916 Spain; Dermatology Service, Hospital de la Santa Creu i Sant Pau, Barcelona, 08040 Spain; Dermatology Service, Hospital Abente y Lago, La Coruña, 15001 Spain; Dermatology Service, Hospital Universitario de Salamanca, Salamanca, 37007 Spain

**Keywords:** Psoriasis, T cell, Gene expression, Microarray, Genetic pathway, Gene network

## Abstract

**Background:**

Psoriasis is a chronic autoimmune disease in which T cells have a predominant role in initiating and perpetuating the chronic inflammation in skin. However, the mechanisms that regulate T cell activation in psoriasis are still incompletely understood. The objective of the present study was to characterize the main genetic pathways associated with T cell activation in psoriasis.

**Results:**

Gene expression profiles from *in vitro* activated T cells were obtained from 17 psoriasis patients and 7 healthy controls using Illumina HT-12 v4 microarrays. From a total of 47,321 analyzed transcripts, 42 genes were found to be differentially expressed between psoriasis and controls (FDR p-value < 0.1, absolute fold-change > 1.2). Using an independent cohort of 8 patients and 8 healthy controls we validated the overexpression of *SPATS2L* (p-value =0.0009) and *KLF6* (p-value =0.0012) genes in activated T cells from psoriasis patients. Using weighted correlation analysis we identified *SPATS2L* and *KLF6* coexpression networks, which were also significantly associated with psoriasis (p-value < 0.05). Gene Ontology analysis allowed the identification of several biological processes associated with each coexpression network. Finally, using Gene Set Enrichment Analysis over the global T cell transcriptome we also found additional genetic pathways strongly associated with psoriasis (p-value < 0.0001).

**Conclusions:**

This study has identified two new genes, *SPATS2L* and *KLF6*, strongly associated with T cell activation in psoriasis. Functional analyses of the gene expression profiles also revealed new biological processes and genetic pathways associated with psoriasis. The results of this study provide an important insight into the biology of this common chronic inflammatory disease.

**Electronic supplementary material:**

The online version of this article (doi:10.1186/1471-2164-14-825) contains supplementary material, which is available to authorized users.

## Background

Psoriasis is a chronic T-cell-mediated inflammatory disease that affects approximately 2% of the world population [1]. Histologically, it is characterized by the infiltration of active immune cells into the skin, the hyperplasia of blood vessels and the hyperproliferation of keratinocytes. The leukocyte infiltrate in psoriasis lesions is composed mainly by T cells, predominantly CD4 + -type cells but also CD8 + -type T cells [2]. T cells are known to expand and migrate into the epidermis before the onset of the first epidermal changes, and steadily continue to do so, promoting and perpetuating the disease [3].

The central role of T cells in psoriasis pathogenesis was first evidenced in the late 70s, when the T cell suppressor cyclosporine A was discovered as an effective treatment for psoriasis inflammation [4]. In the 90′s elegant studies using skin xenografts of with non-lesional skin from patients with psoriasis showed the conversion to psoriatic plaques when mice were injected with preactivated T cells from the same patient [5]. Since then, a substantial amount evidence supporting the key role for T cells, mainly type 1 T helper subtype (Th1), has been accumulated [6–11]. More recently, IL17 and IL22 producing T lymphocytes (Th17 and Th22, respective) have been characterized as critical mediators of psoriasis pathogenesis [12–14]. However, the triggering events and deregulated pathways that lead to the aberrant T cell activation in psoriasis patients are still poorly defined. In the present study we aimed to increase the knowledge on this particular aspect of psoriasis etiology by characterizing the T cell gene expression profile associated with psoriasis during T cell activation.

To date, psoriasis has been investigated by a number of whole genome gene expression studies. These have been mainly conducted with skin biopsy samples, through the comparison of lesional and non-lesional skin. Gene expression analysis in biopsies from skin lesions is of great interest in psoriasis and has produced several important findings [15–21]. However, one limitation of this approach is that the mixture of the different cell types that coexist in the skin tissue is likely to mask relevant gene expression patterns. Specially, if the cell type of interest is not the predominant component of the total tissue extract, as in the case of infiltrating T cells in lesional skin, the resulting gene expression data could not be informative.

An alternative to skin biopsy analysis has been the analysis of gene expression profiles of peripheral blood mononuclear cells (PBMCs) from patients and controls [22–25]. This approach is of particularly interest in immune-mediated diseases like psoriasis, since it is a less invasive approach and, consequently, predictive profiles can have a better translation into the clinical setting. However, most T cells circulating in the peripheral blood are in a quiescent state [26], with scarce gene expression activity [27]. Consequently, relevant pathogenic processes like T cell activation might not well be studied through this approach.

In the present study we have used *in vitro* activated T cells from patients and healthy controls to characterize the gene expression profile associated with psoriasis. Using an independent cohort of patients and controls we have validated the most significant genes associated with this differential regulatory activity. Finally, using different genomic approaches we have characterized the gene expression networks and biological pathways that characterize this key aspect of psoriasis biology.

## Results

Gene expression profiles from *in vitro* activated T cells were obtained from 17 psoriasis patients and 7 healthy controls using Illumina HT-12 v4 microarrays. From each individual gender, age was collected, and Psoriasis Area and Severity Index (PASI) score was determined for each patient at the time of sample collection (Additional file 1). There were no statistically significant differences (P > 0.05) in age or gender between the microarray and validation case control samples. Also, the PASI score was not significantly different between discovery and validation patient samples. From each RNA sample, a total of 47,321 transcripts were quantified and 23,554 probes were called as expressed in the sample (i.e. present in 2 or more individuals) according to the Illumina expression score and selected for statistical analysis.

### Differentially expressed genes in in vitro activated T cells

A total of 42 genes were found to be significantly associated with psoriasis (FDR p-value < 0.1, absolute fold change > 1.2, Additional file 2). From these, 25 were found to be up-regulated in T cells from psoriasis patients and 17 genes were down-regulated in patients compared to controls. The genes with FDR p-value < 0.05 showing the largest change in gene expression (i.e. absolute fold change > 1.2) are shown in Table 1.

**Table 1:** **List of most significantly up-regulated genes in** ***in vitro*** **activated T cells of psoriasis patients compared to healthy controls**

Probe	Gene	Fold change	P value*	Full name
**Upregulated genes**				
ILMN_1683678	***SPATS2L***	1.37	0.0009	Spermatogenesis associated, serine-rich 2-like
ILMN_1735014	***KLF6***	1.32	0.0012	Kruppel-like factor 6
ILMN_1703263	***SP140***	1.38	0.0025	SP140 nuclear body protein
ILMN_2322498	***RORA***	1.31	0.0041	RAR-related orphan receptor A
ILMN_2246956	*BCL2*	1.23	0.0062	B-cell CLL/lymphoma 2
ILMN_2397721	*GLB1*	1.23	0.0062	Galactosidase, beta 1
ILMN_1781285	*DUSP1*	1.21	0.0071	Dual specificity phosphatase 1
ILMN_2321064	*BAX*	1.24	0.0096	BCL2-associated X protein
ILMN_1731107	*CCDC92*	1.33	0.0136	Coiled-coil domain containing 92
ILMN_1729749	*HERC5*	1.72	0.0267	Hect domain and RLD 5
ILMN_2095660	*TMEM156*	1.29	0.0453	Transmembrane protein 156
ILMN_1681301	*AIM2*	1.42	0.0464	Absent in melanoma 2
ILMN_1767470	*SCPEP1*	1.26	0.0469	Serine carboxypeptidase 1
**Downregulated genes**				
ILMN_1778617	*TAF9*	−1.25	0.0009	TAF9 RNA polymerase II, TATA box binding protein (TBP)-associated factor, 32kDa
ILMN_2143250	*FAR1*	−1.20	0.0207	Fatty acyl CoA reductase 1
ILMN_1720114	*GMNN*	−1.20	0.0269	Geminin
ILMN_2135175	*SNORD36A*	−1.27	0.0303	Small nucleolar RNA, C/D box 36A

*Multiple-test corrected p-value.

List of genes with multiple-test corrected p-value < 0.05 and absolute change >1.2. Genes are classified into up-regulated or down-regulated groups and sorted by their statistical significance. Genes that were selected to validate are highlighted in bold.

### Validation of genes associated with psoriasis in T cells

**Figure 1:**
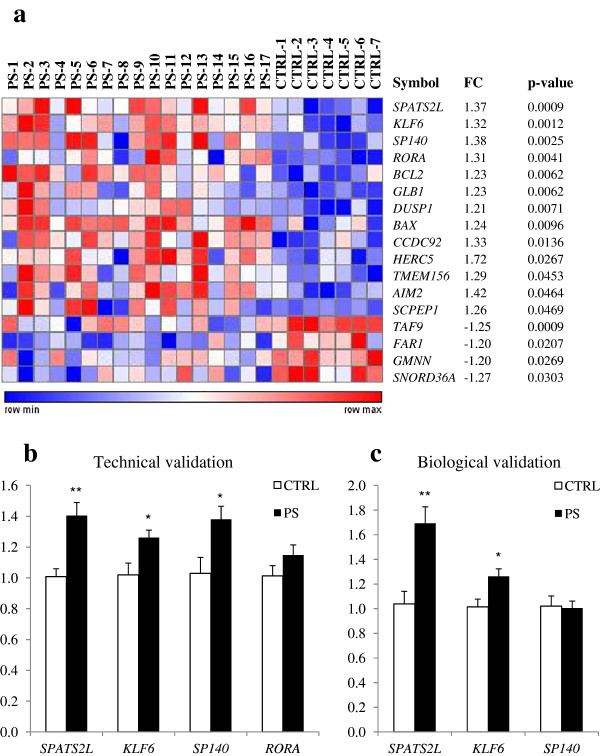
**Validation of the most significantly up-regulated genes in** ***in vitro*** **activated T cells of psoriasis patients compared to healthy controls. (a)** Heat map of microarray expression data for the genes most significantly associated with psoriasis (FDR p-value < 00.5, FCH > 1.2). The gene expression values for each gene have been scaled using the minimum and maximum values in order to represent a normalized gene expression gradient **(b)** Technical validation of *SPATS2L*, *KLF6*, *SP140* and *RORA* genes by RT-PCR (Psoriasis n = 17, and Controls n = 7). **(c)** Biological validation of *SPATS2L*, *KLF6* and *SP140* genes by RT-PCR in an independent cohort of psoriasis patients and healthy controls (n = 8 each). **p < 0.01 and *p < 0.05.

From the list of significant differentially expressed genes, we selected those genes showing a high level of statistical association (Pcorrected < 0.005) with the largest absolute change in expression (i.e. Fold Change (FC) > 1.3) for validation. These genes were spermatogenesis associated serine-rich 2-like (*SPATS2L*, FC = 1.37, Pcorrected = 0.0009), Kruppel-like factor 6 (*KLF6*, FC = 1.32, p-value = 0.0012), *SP140* nuclear body protein (*SP140*, FC = 1.38, p-value = 0.0025) and RAR-related orphan receptor A (*RORA*, FC = 1.31, p-value = 0.0041) (Figure 1a) genes. Using the same set of samples from the microarray analysis we first performed a RT-PCR technical validation study. We were able to validate the differential expression observed for *SPATS2L* (FC = 1.39, p-value = 0.0006), *KLF6* (FC = 1.24, p-value = 0.022) and *SP140* (FC = 1.34, p-value = 0.019) genes (Figure 1b). *RORA* differential expression was not validated (p-value > 0.05) and, consequently, we did not perform biological validation on this sample.

After technical validation, we used an independent sample of patients and controls recruited from the same University Hospitals to validate the association of *SPATS2L*, *KLF6* and *SP140* genes with the activation of T cells in psoriasis. Using *in vitro* activated T cells from 8 patients and 8 controls we significantly validated the overexpression of *SPATS2L* (FC = 1.58, p-value = 0.0014) and *KLF6* (FC = 1.23, p-value = 0.024) genes in psoriasis (Figure 1b). *SP140* did not show significant differential expression in the independent replication cohort.

### Identification of SPAT2SL and KLF6 coexpression networks

Since *SPAT2SL* and *KLF6* have not been previously associated to the T lymphocyte activation process, we aimed to identify and analyze the gene expression network associated to each of the two genes. For this objective we used the weighted correlation analysis implemented in the WGCNA method [28]. After filtering for the most informative genes (i.e. coefficient of variation > 0.1, n = 4,512 genes), we identified a total of 20 different gene expression modules (Additional file 3). Using the eigenvectors derived from each gene expression module we evaluated the association to different clinical and epidemiological variables (Additional file 4). From these, we found four modules significantly associated with the presence of psoriasis (p-value < 0.05, Additional files 5 and 6). These four modules were not associated with any of the other tested covariates. The first of these modules (M1, p-value = 0.00051) included 54 genes and was the most enriched in genes differentially expressed between psoriasis cases and controls (59.3% at nominal p-value < 0.05). The second module (M2, p-value = 0.0038) consisted in 92 genes from which 37% were differentially expressed. The third module (M3, p-value = 0.011) included 31 genes and 29% from them showed differential expression. Finally, the fourth module (M4, p-value = 0.047) included a total of 115 genes, but with a lower percentage showing differential expression between cases and controls (24.3%).

*SPATS2L* was found to belong to M1 gene expression module, while *KLF6* was found to be coexpressed with genes in module M2. The fourth module associated with psoriasis, M4, included the genome-wide significant *RORA* gene that could not be technically validated by RTPCR.

### Gene Ontology analyses of SPATS2L and KLF6 coexpression networks

In order to functionally characterize *SPATS2L* and *KLF6* coexpression networks we performed Gene Ontology (GO) enrichment analysis using DAVID functional annotation tool [29]. Table 2 shows the most significantly enriched GO terms related to biological processes. The complete list, with the significant GO terms associated to cellular component and molecular function, is provided in the Additional file 7.

**Table 2:** **Summary of the enriched GO Biological Process terms in** ***SPATS2L*** **and** ***KLF6*** **coexpression networks**

Biological process in ***SPATS2L*** network			
Term	Count	%	p-value
GO:0051276 ~ chromosome organization	5	12.8	1.90E-02
GO:0042273 ~ ribosomal large subunit biogenesis	2	5.1	2.12E-02
GO:0006461 ~ protein complex assembly	5	12.8	2.17E-02
GO:0070271 ~ protein complex biogenesis	5	12.8	2.17E-02
GO:0042254 ~ ribosome biogenesis	3	7.7	2.79E-02
GO:0034622 ~ cellular macromolecular complex assembly	4	10.3	2.99E-02
GO:0034621 ~ cellular macromolecular complex subunit organization	4	10.3	4.01E-02
GO:0006325 ~ chromatin organization	4	10.3	4.63E-02
**Biological process in** ***KLF6*** **network**			
**Term**	**Count**	**%**	**p-value**
GO:0006695 ~ cholesterol biosynthetic process	5	6.33	3.95E-06
GO:0016126 ~ sterol biosynthetic process	5	6.33	1.35E-05
GO:0044271 ~ nitrogen compound biosynthetic process	8	10.13	4.08E-04
GO:0006694 ~ steroid biosynthetic process	5	6.33	4.45E-04
GO:0008203 ~ cholesterol metabolic process	5	6.33	6.01E-04
GO:0016125 ~ sterol metabolic process	5	6.33	8.54E-04
GO:0009070 ~ serine family amino acid biosynthetic process	3	3.80	9.36E-04
GO:0008652 ~ cellular amino acid biosynthetic process	4	5.06	1.28E-03
GO:0008610 ~ lipid biosynthetic process	7	8.86	2.30E-03
GO:0008299 ~ isoprenoid biosynthetic process	3	3.80	3.16E-03
GO:0009309 ~ amine biosynthetic process	4	5.06	4.80E-03
GO:0006094 ~ gluconeogenesis	3	3.80	4.92E-03
GO:0009069 ~ serine family amino acid metabolic process	3	3.80	5.31E-03
GO:0019319 ~ hexose biosynthetic process	3	3.80	7.50E-03
GO:0070814 ~ hydrogen sulfide biosynthetic process	2	2.53	8.41E-03
GO:0070813 ~ hydrogen sulfide metabolic process	2	2.53	8.41E-03
GO:0019344 ~ cysteine biosynthetic process	2	2.53	8.41E-03
GO:0034976 ~ response to endoplasmic reticulum stress	3	3.80	8.97E-03

List of enriched GO terms related to biological process (BP) in the *SPATS2L* (p-value < 0.05) and *KLF6* coexpression networks (p-value < 0.01). For each term, the number of genes present in the network (count), the percentage of enrichment (%) and the associated p-value are indicated.

In the *SPATS2L* coexpression network, eight GO terms were found to be significantly enriched (p-values from 0.01 to 0.05). Most of these GO terms were related to different aspects of chromosome and ribosomal biology (e.g., GO:0051276, chromosome organization, p-value = 0.019; GO:0042273, ribosomal biogenesis, p-value = 0.021).

In the *KLF6* coexpression network 35 GO terms were found to be significantly enriched (p-values ranging from 3.95×10e-6 to 0.05). In this network, the most significantly enriched GO terms (p-value < 0.01) were involved in lipid synthesis (n = 7 ontologies), followed by GO terms associated with amino acid biosynthesis (n = 6 ontologies), hydrogen sulphide process (n = 2 ontologies) and endoplasmic reticulum stress (n = 1 ontology).

### Gene set enrichment analysis of the T cell transcriptome

In order to further investigate the biological basis of the gene expression differences observed between T cells from psoriasis patients and controls, we analyzed the global transcriptome using the GSEA approach [30, 31]. A total of 55 gene sets were found to be significantly enriched between cases and controls (False Discovery Rate, FDR < 0.05) (Table 3). From these, four gene sets were significant at a FDR < 0.0001 all belonging to Reactome database [32] pathway definitions: Cytokine Signaling in Immune System, Interferon Signaling, Interferon Gamma Signaling and Interferon Alpha/Beta Signaling.

**Table 3:** **Summary of the GSEA-identified gene sets in** ***in vitro*** **activated T cells of psoriasis patients compared to healthy controls**

Name	NES	NOM p-val	FDR q-val
**General processes of immune system**			
Reactome cytokine signaling in immune system	2.498	0.000	0.0000
KEGG cytokine cytokine receptor interaction	1.980	0.000	0.0130
KEGG hematopoietic cell lineage	1.917	0.000	0.0167
KEGG cell adhesion molecules cams	1.903	0.000	0.0173
**Infectious and autoimmune diseases**			
KEGG autoimmune thyroid disease	1.996	0.002	0.0126
KEGG allograft rejection	1.866	0.000	0.0234
KEGG leishmania infection	1.961	0.000	0.0121
KEGG viral myocarditis	1.900	0.000	0.0175
**T cell activation**			
Reactome downstream TCR signaling	1.869	0.000	0.0232
PID NFAT TF pathway	1.903	0.000	0.0179
PID AP1 pathway	1.936	0.000	0.0148
Biocarta CTLA4 pathway	1.854	0.000	0.0253
**Innate immune system activation**			
Reactome innate immune system	2.145	0.000	0.0014
Reactome inflammasomes	1.905	0.000	0.0180
Reactome antiviral mechanism by IFN stimulated genes	1.915	0.000	0.0160
Reactome RIG I MDA5 mediated induction of IFN alpha beta pathways	2.212	0.000	0.0005
KEGG RIG I like receptor signaling pathway	2.144	0.000	0.0012
Reactome negative regulators of RIG I MDA5 signaling	2.123	0.000	0.0018
KEGG NOD like receptor signaling pathway	1.953	0.000	0.0128
Reactome nucleotide binding domain leucine rich repeat containing receptor NLR signaling pathways	2.048	0.000	0.0058
KEGG toll like receptor signaling pathway	1.802	0.000	0.0398
Reactome TRAF6 mediated IRF7 activation	1.875	0.002	0.0226
KEGG cytosolic DNA sensing pathway	1.781	0.002	0.0449
**Cytokine signaling**			
KEGG JAK STAT signaling pathway	1.977	0.000	0.0119
PID IL12 STAT4 pathway	1.775	0.004	0.0454
Reactome interferon signaling	2.701	0.000	0.0000
Reactome interferon alpha beta signaling	2.760	0.000	0.0000
Reactome interferon gamma signaling	2.303	0.000	0.0000
PID IFNG pathway	1.787	0.002	0.0434
PID SMAD2 3 nuclear pathway	2.094	0.000	0.0031
Reactome transcriptional activity OF SMAD2 SMAD3 SMAD4 heterotrimer	1.794	0.002	0.0421
Reactome SMAD2 SMAD3 SMAD4 heterotrimer regulates transcription	1.771	0.012	0.0451
**Differentiation, proliferation and apoptosis**			
PID notch pathway	1.916	0.000	0.0163
PID Betacatenin Nuc pathway	1.844	0.002	0.0271
Biocarta gleevec pathway	1.862	0.000	0.0237
PID CMYB pathway	1.842	0.000	0.0271
Reactome YAP1 AND WWTR1 TAZ stimulated gene expression	1.804	0.008	0.0400
PID P53 downstream pathway	1.772	0.000	0.0452
**Metabolism**			
Reactome cholesterol biosynthesis	2.050	0.000	0.0063
Reactome fatty acid triacylglycerol and ketone body metabolism	1.961	0.000	0.0128
Reactome metabolism of lipids and lipoproteins	1.927	0.000	0.0157
KEGG biosynthesis of unsaturated fatty acids	1.790	0.006	0.0429
KEGG glycine serine and threonine metabolism	1.877	0.000	0.0227
KEGG metabolism of xenobiotics by cytochrome P450	1.924	0.002	0.0157
Reactome PPARA activates gene expression	2.076	0.000	0.0043
Biocarta PPARA pathway	1.780	0.006	0.0444
**Unfolded protein response**			
Reactome unfolded protein response	1.972	0.000	0.0120
Reactome perk regulated gene expression	1.872	0.000	0.0230
Reactome activation of genes by ATF4	1.896	0.000	0.0180
PID ATF2 pathway	1.977	0.000	0.0125
**Other**			
KEGG intestinal immune network for IGA production	1.996	0.000	0.0117
KEGG lysosome	1.951	0.000	0.0127
Reactome generic transcription pathway	1.813	0.000	0.0372
PID HIF1 TFPATHWAY	1.778	0.002	0.0444
Reactome RORA activates circadian expression	1.773	0.007	0.0457

List of the enriched gene sets in psoriasis phenotype compared to control phenotype showing a FDR < 0.05. Gene sets are classified according to the processes where they are involved. Normalized enrichment score (NES), nominal p-value (NOM p-value) and false discovery rate (FDR q-value) are indicated.

The top gene sets identified using GSEA encoded for cytokine signalling-related genes, more specifically, for genes associated with alpha/beta and gamma interferon signalling. Consistent with this strong interferon response, a group of gene sets related to innate immune system (FDR = 0.0014) and RIG and NOD-like receptor pathways (FDR = 0.0005 and 0.0058, respectively) were also among the most significantly enriched.

T cell activation in psoriasis patients also resulted in a significant enrichment of genes involved in PPARα-mediated activation of gene expression (FDR = 0.0043) and cholesterol biosynthesis (FDR = 0.0063). Cholesterol biosynthesis was also the most significant GO biological process associated with *KLF6* coexpression network. Finally, protein misfolding in the endoplasmic reticulm was also a common pathway detected by GSEA (i.e. unfolded protein response, FDR = 0.012) and in the GO analysis of *KLF6* network.

## Discussion

It is widely accepted that psoriasis is a T-cell mediated disease, in which lymphocytes continuously infiltrate the skin promoting and perpetuating inflammation. However, little is known yet on the gene expression regulation that characterizes T cell activation in psoriasis. Most of the whole genome gene expression studies in psoriasis performed to date have been principally been conducted in skin, where the T cell-associated gene expression is obscured by other more predominant cell types like hyperproliferative keratinocytes. Alternatively, in gene expression studies using freshly isolated blood lymphocytes, most T cells are found in a quiescent state and might not reflect gene expression profiles relevant to the disease. In the present study, we aimed to overcome these limitations by characterizing the differential gene expression profile of *in vitro* activated T cells in psoriasis patients and controls. To our knowledge, it is the first study to analyze T cell activation in psoriasis using whole-genome gene expression analysis.

In the microarray analysis of *in vitro* activated T cells we were able to identify 42 genes differentially expressed between psoriasis patients and controls. Using an independent sample of patients and controls we validated the association of the two genes most highly overexpressed in psoriasis T cells, *SPATS2L* and *KLF6*. It is the first time that *SPATS2L* and *KLF6* genes have been associated with the T cell activation process in psoriasis.

To date, little is known on the biological functionality of *SPATS2L*. In our study, combining gene coexpression network analysis and Gene Ontology enrichment we found that *SPATS2L* gene network is associated to processes associated with the activation of gene expression including chromosomal organization and ribosomal biogenesis. This potential association of *SPATS2L* with ribosomal processes and translational control has been previously described in gene-trap experiments conducted in myoblasts under oxidative stress [33]. Ribosomal biogenesis has been proved to be critical in the control of cell growth and cell proliferation [34], and in T cells, is one of the most significantly up-regulated cellular processes during cell-cycle entry [35]. Therefore, the overexpression of *SPATS2L* in activated T cells from psoriasis patients could be contributing to an earlier activation of these lymphocytes compared to healthy controls.

Consistently with this potential role in altered cell activation, there is evidence that *SPATS2L* is associated with lymphoproliferative-type of disorders. Analyzing the overexpression of *SPATS2L* in different human diseases, we found that *SPATS2L* is one of the genes most significantly overexpressed in lymphocytes from patients with chronic lymphocytic leukemia (CLL) compared to cells from normal controls (Additional file 8) [36]. Also, *SPATS2L* is found to be significantly overexpressed in a mouse model of T-cell acute lymphoblastic leukemia (Additional file 8) [37]. Furthermore, gene expression data from leukemia cell lines treated with anti-leukemic agent Imatinib, shows a significant downregulation of *SPATS2L* (Additional file 8). Finally, a recent genome-wide association study (GWAS) data on risk loci for lymphoblastic leukemia has shown a significant association of a Single Nucleotide Polymorphism in the *SPATS2L* locus [38]. Together, these results show that *SPATS2L* functionality is strongly associated with the aberrant activation of lymphocytes.

Interestingly, there is evidence that psoriasis and lymphoproliferative disorders could share common genetic basis. Patients with psoriasis have an increased risk of developing leukemia, in particular lymphoma [39]. In a GWAS performed in follicular lymphoma, the region with most significant association was located near the psoriasis susceptibility region 1 (PSORS1) in chromosome 6 [40]. Furthermore, a fairly common manifestation of certain leukemias, including lymphocytic leukemias, is the infiltration of neoplastic leukocytes in skin tissue, also known as leukemia cutis [41]. Therefore, it is tempting to speculate that *SPATS2L* is involved in a common genetic pathway associated with psoriasis and lymphoproliferative-type of disorders. This pathway would therefore participate in the aberrant lymphocyte activity observed in both diseases and the preferential infiltration of skin tissue by these altered lymphocytes.

*KLF6* encodes a member of Kruppel family of transcription factors [42], several of which have been previously implicated in T lymphocyte biology [43]. *KLF6* has been previously shown to be expressed in lymphoid cells [44, 45] and, similarly to *SPATS2L*, there is also evidence of differential expression of *KLF6* in T cells from CLL patients compared to controls [46]. Compared to *SPATS2L* however, *KLF6* coexpression network showed a highly significant Gene Ontology enrichment, mainly in ontologies related to the biosynthesis of lipids and proteins. These biosynthetic processes are induced in cells that are stimulated to rapidly grow and proliferate, as it is the case of activated T cells [47–50]. Two other significant biological processes enriched in the *KLF6* network were endoplasmic reticulum stress and biosynthesis of hydrogen sulfide. These processes are linked to another process also found to be enriched by psoriasis in GSEA analysis which is the unfolded protein response (UPR) [51, 52]. Interestingly, the expression of *KLF6* and other KLF members has been previously reported to be up-regulated when UPR is induced by different drugs, such as tunicamycin [53] or thapsigargin (Additional file 8) [54]. On the basis of this information, it is possible that in T cells from psoriasis patients, *KLF6* up-regulation may also be induced by an UPR, in this case, triggered by the increase in protein-folding load required upon T cell activation.

We have investigated the biological processes that take place during T cell activation associated with psoriasis using both an unsupervised approach which does not use sample information, WGCNA, as well as a supervised approach, GSEA, which compares gene sets overrepresented in cases compared to controls. Even though these are clearly different functional analysis, they resulted in the identification of common biological processes. One of these common biological processes was the biosynthesis of lipids and proteins. For example, cholesterol biosynthesis was the most significantly enriched process in *KLF6* coexpression network and was also detected by GSEA as one of the most enriched processes in T cells from psoriasis patients. This enrichment in genes involved in biosynthetic processes could be attributed to an early activation of T cells from psoriasis patients. Another common biological pathway identified in both approaches was the endoplasmic reticulum stress and unfolded protein response. Interestingly, these two processes, which may result from the increased protein-folding load required in activated T cells, have been associated with a number of chronic inflammatory and autoimmune diseases like spondyloarthritis, rheumatoid arthritis, inflammatory bowel diseases or multiple sclerosis [55–57].

The most significantly enriched gene sets identified by GSEA were, however, related to cytokine signalling (FDR < 0.0001), more specifically, to genes involved in the interferon signalling pathway (IFN pathway FDR < 0.0001; IFNG signalling, FDR < 0.0001; IFNA/B signalling, FDR < 0.0001). This result is fully consistent with the strong interferon-signature detected in psoriasis skin lesions [20]. In line with this observation, GSEA results also show evidence of an association with IL12-STAT4 pathway, an interferon gamma inducing pathway, although at much lower significance. IFN has been shown to drive activation and differentiation of IL17 and IL22 producing T cells [58], and to increase keratinocyte responsiveness to IL22 which leads to the observed hyperproliferation [59]. In line with these observations, GSEA analysis also identified an enrichment of innate immune sensing mechanisms related to the production of IFN like RIG-I receptor signaling pathway (FDR = 0.0005) [60] and the NLR family of receptors like NOD2 [61] (FDR = 0.0058). Together, these results suggest that IFN response pathway is more strongly activated in T cells from psoriasis patients than normal healthy controls.

The four T cell gene networks associated with psoriasis risk identified through WGCNA analysis were found to be highly enriched for genes associated to the response to virus biological process (Additional file 9). A total of 14 genes (*BCL2*, *ISG15*, *EIF2AK2*, *IFIH1*, *IRF7*, *ISG20*, *IFI35*, *IFI44*, *MX1*, *MX2*, *RSAD2*, *STAT1, STAT2* and *TRIM5*) out of 61 overexpressed by T cells (P < 1e-16, test for enrichment) were associated to the response to a viral infection. This result is in accordance with the recent evidence implicating an increased antiviral activity in psoriatic skin compared to skin from normal controls or patients with atopic dermatitits [62]. In this model, keratinocytes are rendered hyperresponsive towards an antiviral response after being conditioned from T helper 17 (Th17) cell-type cytokines.

In the last years the two newly characterized T-cell subsets, Th17 and Th22, have been increasingly implicated in the pathogenesis of psoriasis. While our approach does not provide direct evidence of an increase of either cell subset, it describes an increased activation of precursor cells in affected individuals. It is therefore likely that this differential activity predisposes to these two particular cell types once in the skin microenvironment of psoriasis patients. Additional studies will be needed to confirm this hypothesis. Another limitation of our study is that we analyzed the global expression changes of all activated T cells, regardless of the specific subphenotype. While we found no statistically significant differences in CD4+ or CD8+ percentages in cases compared to controls (data not shown), it is possible that specific subphenotypes are differently represented between the two groups. Therefore, future studies will be performed to identify which cell subtype/s are responsible for each of the differentially expressed genes and genetic pathways associated with psoriasis in our study.

## Conclusions

In the present study, we have investigated for the first time the gene expression profile specifically associated with T cell activation in psoriasis. We have identified *SPATS2L* and *KLF6* as the two genes most significantly overexpressed in activated T cells from psoriasis patients and we have validated this overexpression in an independent case-control cohort. Functional analysis of the gene coexpression networks, suggest that *SPATS2L* and *KLF6* might be involved in an early activation process. Global functional enrichment also shows a strong prevalence of interferon cytokine mediated signalling in activated T cells from psoriasis compared to controls. The results of this study associate new biological pathways with psoriasis etiology and establish new lines of research that will clearly lead to an improved knowledge of the biological basis of this disease and, hopefully, to improved therapies in the near future.

## Methods

### Subjects

Fifteen healthy controls and twenty-five psoriasis patients were included in the present study. Controls (mean ± SD age 40.8 ± 8.8 years; 9 men and 6 women) didn’t have psoriasis or any other immune mediated inflammatory disease and were collected from medical staff at the Vall d’Hebron Hospital (Barcelona, Spain). Psoriasis patients (mean ± SD age 46 ± 8.4 years; 14 men and 11 women), were recruited from the outpatient’s clinics of the dermatology departments from Santa Creu i Sant Pau Hospital (Barcelona, Spain) and Germans Trias i Pujol Hospital (Badalona, Spain). All selected patients had chronic plaque psoriasis as diagnosed by a dermatologist, affecting torso and/or extremities with at least one year of duration. Patients with a single clinical localization of plaque psoriasis (i.e. scalp, face, palmoplantar), with exclusively inverse plaque psoriasis or with an inflammatory bowel disease were excluded. All patients were >18 years old at the time of recruitment, although disease onset could have occurred at an earlier age.

Informed consent was obtained from all participants and protocols were reviewed and approved by the Vall d’Hebron University Hospital ethics committee. The present study was conducted according to the Declaration of Helsinki principles. Additional file 1 summarizes the main clinical features of patients and controls (age, gender, PASI score).

### Cell culture

Peripheral blood mononuclear cells (PBMCs) were isolated from whole blood of patients and controls by centrifugation on a Ficoll-Paque PLUS (GE Healthcare Biosciences AB, Uppsala, Sweden) density gradient. PBMCs were then seeded in complete RPMI 1640 medium supplemented with 100 U/ml penicillin, 100 U/ml streptomycin, 10% fetal bovine serum, 2 mM L-glutamine and 50 μM mercaptoethanol, and activated with 1μg/ml of anti-CD3 and anti-CD28 antibodies (eBioscience, San Diego, CA, USA). After 72 hours of stimulation, activated T cells were harvested by centrifugation and processed for flow cytometry study or frozen at -20°C for subsequent RNA isolation.

### Flow cytometry analysis

In order to characterize the T lymphocyte population present in cultured PBMCs samples and verify their activation after stimulation by CD3 and CD28 ligation, we performed flow cytometric analysis. *In vitro* activated cells were stained with anti-human CD4-PE (eBioscience, San Diego, CA, USA) and anti-human CD8-PerCP (Biolegend, San Diego, CA, USA) antibodies before acquisition (FC500 Flow Cytometer, Beckman Coulter, CA, USA) and analysis (Flowjo, Tree Star, Inc., Oregon, USA). Forward and side scatters were used to gate resting and blast lymphocytes, and CD4+ or CD8+ -type cells were counted within each gate. The average percentage of blasting cells was 75.94 (+/- 9.9) with 51.36% (+/-11.3) being CD4+ and 37.87% (+/-10.7) corresponding to CD8+. No statistically significant differences were observed between cases and controls.

### RNA isolation

Total RNA was isolated from previously activated T cells using the RNeasy mini kit (Qiagen, Hilden, Germany). RNA yield was assessed using a NanoDrop 1000 spectrophotometer (Thermo Fisher Scientific, USA) and RNA integrity was assessed using the RNA 6000 Nano LabChip Kit on an Agilent 2100 Bioanalyser (Agilent technologies, Waldbronn, Germany). All RNA samples had a RNA Integrity Number higher than 9 and were subsequently used for microarray analysis.

### Microarray analysis and differential gene expression determination

Whole genome gene expression analysis of *in vitro* activated T cells for 17 psoriasis patients and 7 healthy controls was carried out by the HudsonAlpha Institute for Biotechnology using the Illumina HT-12 V4 microarrays (Illumina, USA). Briefly, the Illumina CustomPrep RNA amplification kit (Appiled Biosystems, USA) was used to generate biotinylated amplified complementary RNA (cRNA) from 200 ng of the extracted total RNA. Labelled cRNA was then hybridized overnight to the Illumina microarrays, washed, blocked, stained and scanned using the Illumina BeadStation 500. The GenomeStudio software v2011.1 (Illumina, USA) was used to generate the raw data from the microarray scanned images. HT-12 V4 microarrays allow the simultaneous profiling of >47,000 transcripts, which include 28,688 well-established RefSeq annotations from the Human RefSeq (Rel 38). Sample labeling, microarray hybridization, scanning and raw data acquisition were performed at HudsohAlpha Institute for Biotechnology (Alabama, USA).

Data preprocessing, normalization and differential expression was performed using the R-statistical software v12.0 [63]. Log2-transformed data was normalized using the quantile normalization method [64]. Using the probe detection p-value calculated by GenomeStudio, only those transcripts present in 3 or more samples were considered for differential expression analysis. After filtering, a total of 25,539 transcripts were finally used to determine the associated gene expression profiles. Differential gene expression between cases and controls was tested using the *t*-test method implemented in the Bioconductor multtest package [65]. Correction for multiple testing was performed using the Benjamini and Hochberg False Discovery Rate method [66]. From these significant genes, only those having an absolute fold change >1.2 were considered to be differentially expressed between the two conditions.

### Real-time PCR validation

200 ng of total RNA from 8 psoriasis cases and 8 controls were reverse-transcribed using the High Capacity cDNA Reverse Transcription Kit (Invitrogen, Carlsbad, CA) following the manufacturer’s instructions. Real-time PCRs were performed on an ABI PRISM7900 Sequence Detection HT system (Applied Biosystems, Foster City, CA, USA) and using the TaqMan® chemistry (Applied Biosystems, Foster City, CA, USA). The thermal cycle conditions were: 50°C for two minutes, 95°C for 10 minutes and 40 cycles of 95°C for 15 seconds and 60°C for one minute. The selected TaqMan expression assays were: Hs01016366_m1 (*SPATS2*), Hs00296661_s1 (*KLF6*), Hs00916867_m1 (*SP140*), Hs00931153_m1 (*RORA*) and Hs01060665_g1 (*ACTB*). The method of 2 - [Delta] [Delta] Ct was used to obtain the mRNA expression of psoriasis samples relative to the control samples, normalizing the Ct values of target genes with those of the housekeeping gene actin beta (*ACTB*).

### Gene expression network analysis

Genes involved in a same biological process or genetic pathway tend to show correlated levels of expression. Using this property there are different methods that are able to build up networks of genes that better characterize the biological process of interest [67]. Such analysis can also provide a powerful approach for elucidating the unknown role of a given gene, based on the pathways or processes in which the other genes in the same network are involved. In the present study we have used the Weighted correlation network analysis (WGCNA) approach to infer the gene expression modules in activated T cells that are associated with psoriasis [28]. After calculating the co-expression matrix using Pearson’s correlation method, a network adjacency is calculated by raising the co-expression similarity to a power β. Then using the scale-free topology criterion [68], the threshold parameter that defines the connectivity between genes is chosen. Once the threshold for connectivity is set, the coexpression network can be built. Within this network, clusters of densely connected genes (i.e. modules) are defined using unsupervised clustering and the Dynamic Tree Cut method [69]. Finally, for each gene expression module a summary measure or eigengene can be calculated using the first principal component of the corresponding expression matrix. Each eigengene was subsequently used to test for association with psoriasis and other relevant clinical and epidemiological covariates (age, gender, PASI score, % of activated lymphocytes and % CD4 or CD8 subsets). For binary variables the *t*-test was used, while for quantitative variables a linear regression approach was used.

In order to contextualize the gene networks associated with psoriasis, we performed a comprehensive search for previous studies having found evidence between the network genes and the disease. Using each gene annotation and the term ‘psoriasis’ we performed systematic queries using NCBI Pubmed database (October 2013). In order to reduce the presence of false positives only genes with absolute FCH > 1.2 were included. A total of 70 overexpressed genes and 22 underexpressed genes were queried.

### Functional annotation of expression modules

Gene Ontology (GO) category enrichment analysis was performed using DAVID online tool (i.e. Database for Annotation, Visualization and Integrated Discovery, http://david.abcc.ncifcrf.gov/, Bethesda, MD, USA) [29]. Briefly, GO enrichment is calculated comparing the frequency of genes from each different GO present in the set of differently expressed genes to the frequency of genes for this same GO in the human genome background.

### Gene set enrichment analysis

GSEA is a software method to analyze and interpret microarray data using biological knowledge [30, 31]. Using the differential gene expression result between cases and controls and a list of predefined biological sets of genes, GSEA tests the overrepresentation each gene set in the group of most associated genes compared to the group of genes showing lower –or none- evidence of association. In the present study we used GSEA version 2.0.12 (www Broad Institute at MIT), with the C2 curated canonical pathway gene set (i.e. c2.cp.v3.1.symbols.gmt download), collapsing of dataset to gene symbols, weighted enrichment statistic and real gene list sorting. Maximum size of gene sets was set to 500 genes and minimum gene set size was set to 15. Empirical significance values were calculated using 10,000 permutations.

### Availability of supporting data

The datasets supporting the results of this article are available in NCBI’s gene Expression Omnibus and are accessible through GEO Series accession number GSE47598 (http://www.ncbi.nlm.nih.gov/geo/query/acc.cgi?acc=GSE47598).

## Electronic supplementary material

Additional file 1: **Data on the subjects included in the study.** List of subjects included in the microarray and validation studies with information relative to their gender, age and PASI score. M, male; F, female; PASI, Psoriasis Area Severity Index. (XLS 12 KB)

Additional file 2: **Differentially expressed genes in** ***in vitro*** **activated T cells.** Complete list of genes with a Benjamini and Hochberg corrected p-value < 0.1 and absolute FCH > 1.2. Genes are classified in up-regulated or down-regulated and sorted by their statistical significance. (XLS 34 KB)

Additional file 3: **Gene coexpression networks in** ***in vitro*** **activated T cells.** 20 gene coexpression modules that resulted from weighted correlation network analysis applied to the complete microarray samples. (XLS 623 KB)

Additional file 4: **Association results of the gene coexpression modules with different clinical and epidemiological variables.** For binary variables the *t*-test was used, while for quantitative variables a linear regression approach was used. Significant p-values (P < 0.05) are highlighted in bold. (XLS 24 KB)

Additional file 5: **Visualization of the gene modules associated with psoriasis.** Using a heatmap plot we can visualize the level of adjacency of the genes that conform the four gene expression networks associated with psoriasis (light colors indicate low adjacency, dark colors high adjacency). Each module is depicted by a different color: M1 (grey), M2 (green), M3 (blue) and M4 (pink). (PDF 471 KB)

Additional file 6: **Visualization of the eigengene network with each clinical variable.** Using a heatmap plot we can visualize the relationships among the modules (representetd by their eigengenes) and each of the clinical traits. The level of correlation is a scale that goes from 0 (green) to 1 (red). Each module is depicted by a different color: grey (M1), greenyellow (M2), royalblue (M3), pink (M4), black (M5), blue (M6), brown (M7), cyan (M8), green (M9), lightcyan (M10), lightgreen (M11), lightyellow (M12), magenta (M13), midnightblue (M14), purple (M15), red (M16), salmon (M17), tan (M18), turquoise (M19), yellow (M20). (PDF 22 KB)

Additional file 7: **Summary of enriched GO terms in** ***SPATS2L*** **and** ***KLF6*** **coexpression networks.** List of enriched GO terms related to biological process, cellular component and molecular function in the *SPATS2L* and *KLF6* coexpression networks. For each term, the number of genes present in the network (count), the percentage of enrichment (%), the associated p-value and the genes present in the network are indicated. (XLS 34 KB)

Additional file 8: **List of GEO profiles showing highly significant differential expression of** ***SPATS2L*** **and** ***KLF6*** **genes.** Selection of *SPATS2L* and *KLF6* GEO profiles showing highly significant differential expression. For each GEO profile, the accession number, the title and a description of their corresponding GEO DataSets are provided. (XLS 25 KB)

Additional file 9: **List of PubMed articles on genes from the T cell gene networks associated with psoriasis risk.** (XLS 37 KB)
