# Supplementary figures and images for: Genome-wide transcriptional analysis of T cell activation reveals differential gene expression associated with psoriasis
Source: BMC Genomics. 2013 Nov 23;14(1):825. doi: 10.1186/1471-2164-14-825 (PMC4046651; doi:10.1186/1471-2164-14-825)

Network heatmap plot

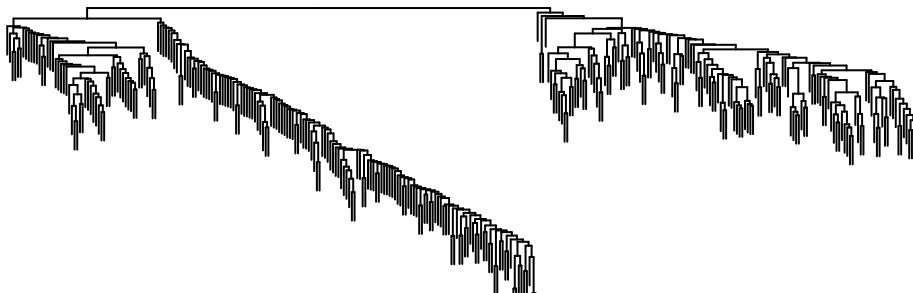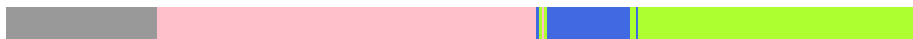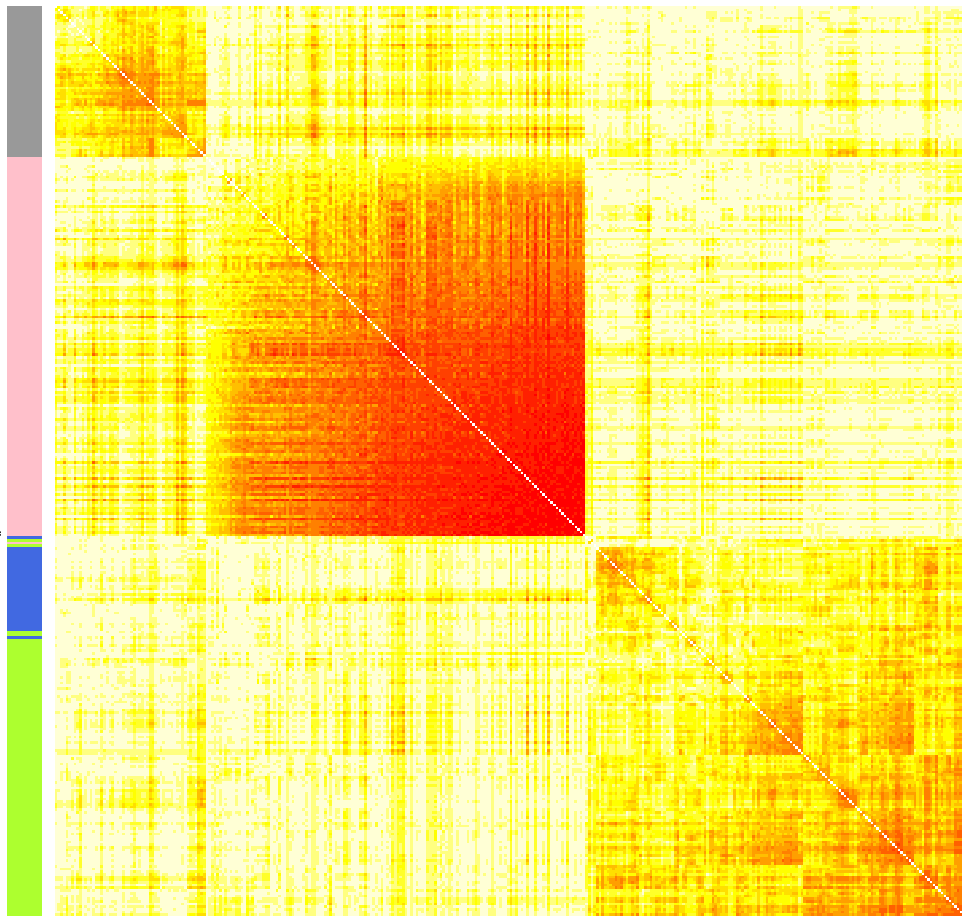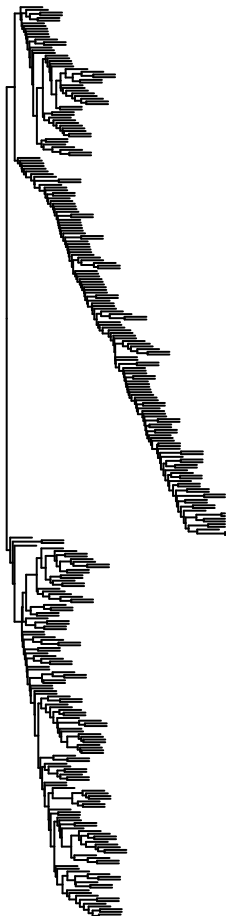

Supplement: Supplementary file 5 — Additional file 5: Visualization of the gene modules associated with psoriasis. Using a heatmap plot we can visualize the level of adjacency of the genes that conform the four gene expression networks associated with psoriasis (light colors indicate low adjacency, dark colors high adjacency). Each module is depicted by a different color: M1 (grey), M2 (green), M3 (blue) and M4 (pink). (PDF 471 KB) [file 12864_2013_5528_MOESM5_ESM.pdf]

Eigengene heatmap: Disease

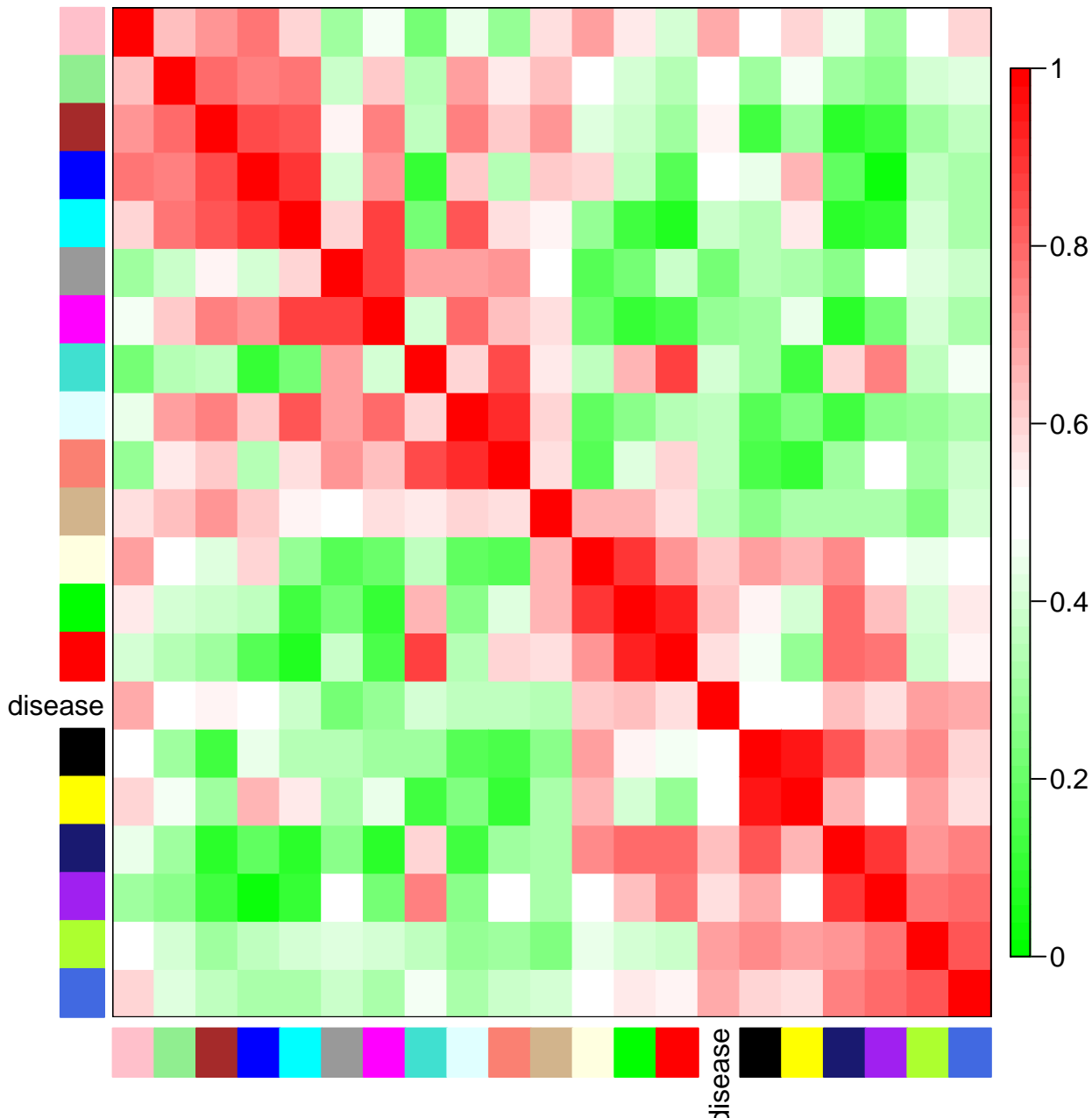

Eigengene heatmap: Sex

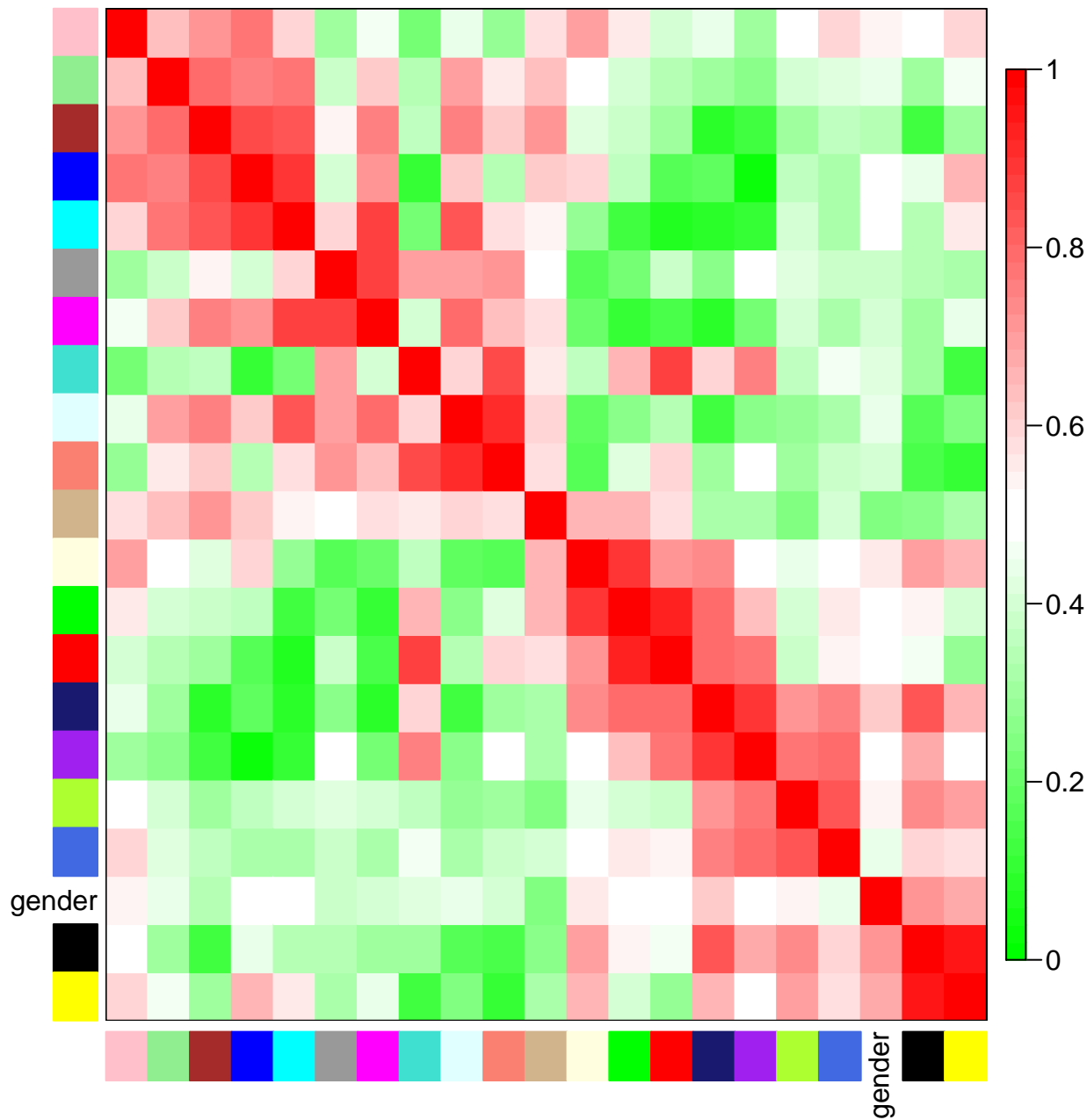

[illegible]

Supplement: Supplementary file 6 — Additional file 6: Visualization of the eigengene network with each clinical variable. Using a heatmap plot we can visualize the relationships among the modules (representetd by their eigengenes) and each of the clinical traits. The level of correlation is a scale that goes from 0 (green) to 1 (red). Each module is depicted by a different color: grey (M1), greenyellow (M2), royalblue (M3), pink (M4), black (M5), blue (M6), brown (M7), cyan (M8), green (M9), lightcyan (M10), lightgreen (M11), lightyellow (M12), magenta (M13), midnightblue (M14), purple (M15), red (M16), salmon (M17), tan (M18), turquoise (M19), yellow (M20). (PDF 22 KB) [file 12864_2013_5528_MOESM6_ESM.pdf]
